# Estimating the Potential Health Care Cost-Savings from a Flax-Based Treatment for Hypertension

**DOI:** 10.3390/nu16162638

**Published:** 2024-08-10

**Authors:** Luc Clair, Jared Kashton, Grant N. Pierce

**Affiliations:** 1 Canadian Centre for Agri-Food Research in Health and Medicine, Albrechtsen Research Centre, St. Boniface Hospital, Winnipeg, MB R2H 2A6, Canada; kashtonjared@gmail.com (J.K.); gpierce@sbrc.ca (G.N.P.); 2 Department of Economics, Faculty of Business and Economics, University of Winnipeg, Winnipeg, MB R3B 2E9, Canada; 3 Institute of Cardiovascular Sciences, Albrechtsen Research Centre, St. Boniface Hospital, Winnipeg, MB R2H 2A6, Canada; 4 Department of Physiology and Pathophysiology, Rady Faculty of Health Sciences, University of Manitoba, Winnipeg, MB R3E 0W3, Canada

**Keywords:** hypertension, cost-of-illness analysis, flaxseed

## Abstract

Hypertension contributes to the increase in health care spending in Canada through two primary mechanisms. First, it directly increases costs, as individuals with hypertension require medical care to manage the condition. Second, it indirectly raises expenses by serving as a risk factor for numerous chronic diseases, leading to increased health care utilization among those affected. Therefore, reducing hypertension prevalence could alleviate its resulting strain on the Canadian health care system. Clinical trials have demonstrated that daily flaxseed consumption effectively lowers both systolic and diastolic blood pressure. This study employs a four-step cost-of-illness analysis to estimate the potential health care cost-savings from a flaxseed-based treatment for hypertension. The analysis begins by assessing the proportion of individuals with hypertension likely to adopt the flaxseed regimen. It then evaluates the impact of flaxseed consumption on systolic and diastolic blood pressure. Next, data from the Canadian Health Measures Survey, Cycles 5 and 6, are used to estimate the prevalence of hypertension and the expected reduction in prevalence due to the flaxseed treatment. Finally, the potential reduction in health care spending is calculated. To incorporate uncertainty, partial sensitivity analysis and Monte Carlo simulations were utilized, varying the intake success rate and other model parameters, respectively. The most conservative estimate suggests a potential health care cost-savings of CAD 96,284,344 in Canada for the year 2020.

## 1. Introduction

**Figure 1:**
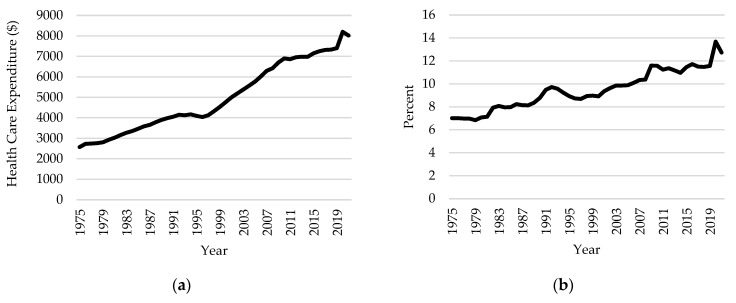
Trends in health care spending in Canada, 1975–2021 (source: Canadian Institute of Health Information National Health Expenditure Trends). (**a**) Total health expenditures per capita (Constant 2021 CAD). **(b)** Health care spending as percentage of gross domestic product.

**Figure 2:**
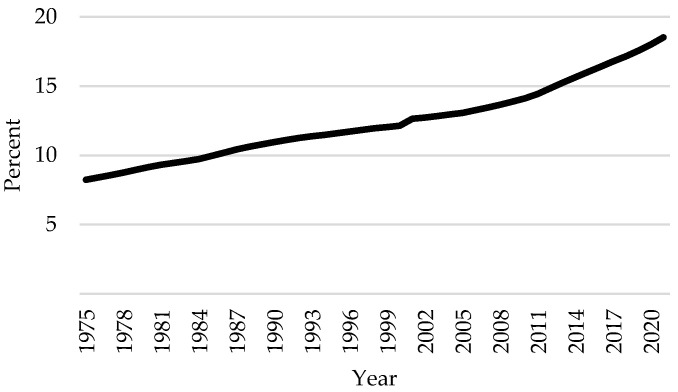
Share of Canadian population 65 years or older (1975–2021).

Health care spending in Canada has been growing steadily over the past few decades, reaching an estimated CAD 308 billion in total expenditure in 2021 [1]. This is equivalent to CAD 8019 per Canadian and 12.7% of the GDP (See Figure 1). This rise is primarily driven by Canada’s changing demographics [2]. The proportion of Canadians 65 years old or older grew by more than ten percentage points between 1975 and 2021 (See Figure 2), a trend expected to continue as members of the baby boom generation enter this age cohort. As the senior population expands, so does the prevalence of chronic diseases such as cardiovascular disease (CVD), dementia, diabetes, renal disease, and cancer [2,3,4]. Furthermore, Canada’s health care system is better designed to treat acute illnesses and often employs high-cost resources to treat chronic diseases [5]. Thus, finding ways to mitigate the incidence and prevalence of chronic diseases is crucial for reducing societal health care costs.

One chronic disease that serves as a precursor for many of these illnesses is hypertension [6,7,8,9,10]. Hypertension is a sustained increase in an individual’s blood pressure, which can weaken blood vessels and increase the risk for arteriosclerosis. Therefore, hypertension is a costly illness, as it indirectly contributes to health care utilization through the development of more severe forms of CVD. In addition, the direct costs of treating hypertension can be staggering. An estimated CAD 20.5 billion in health care spending was attributed to hypertension in 2020, approximately 7% of the total expenditure in that year [1,11]. Given Canada’s aging population, it can be reasonable to expect that the burden of hypertension will increase over the coming years.

Recent studies have shown that functional foods containing flaxseed can effectively reduce BP, with greater efficacy observed in individuals with more severe hypertension [12,13,14,15]. The mechanism by which flaxseed interferes with blood pressure has been studied previously. Flaxseed, through its rich alpha-lipoic acid (ALA) content, inhibits the generation of pro-inflammatory compounds, and inflammation is known to contribute to CVDs such as hypertension [16,17,18,19]. More directly, with regard to hypertension, the ALA in flaxseed has been shown to inhibit soluble epoxide hydrolase, a key enzyme in oxylipin metabolism that alters the generation of both inflammatory molecules and vasodilators [13,18]. Through the inhibition of soluble epoxide hydrolase, the ALA in flaxseed inhibits the generation of pro-inflammatory molecules and enhances the generation of vasodilators to effectively lower BP [13,18]. Soluble epoxide hydrolase has also been identified as a key target of pharmaceutical trials in an effort to lower BP [20]. Additionally, Health Canada has claimed dietary flaxseed to be an effective method for lowering low-density lipoprotein (LDL) cholesterol and total plasma cholesterol concentrations, which may contribute to lower blood pressure by improving overall cardiovascular health [21,22]. Recent research further suggests that the increased consumption of flaxseed may improve overall quality of life and reduce the number of disability-adjusted life years [23]. Hence, using dietary flaxseed to help treat hypertension may help alleviate the financial burden associated with the disease.

This study aims to estimate the potential health care cost-savings obtained from a flax-based treatment for hypertension. We perform a modified four-step cost-of-illness (COI) analysis using data from the Canadian Health Measures Survey (CHMS). As part of our analysis, we examine the systolic blood pressure (SBP), diastolic blood pressure (DBP), and the prevalence of hypertension in Canada for individuals 18 years of age and older. Using partial sensitivity analysis and Monte Carlo simulations, we find that the potential annual health care cost-savings obtained from the flax-based treatment for hypertension range from CAD 96,284,343 to CAD 985,145,703 in 2020.

## 2. Materials and Methods

### 2.1. Cost-of-Illness Analysis

To estimate the potential health care cost-savings from the flax-based treatment for hypertension, we conduct a modified four-step COI analysis, as outlined in the work of Abdullah et al. (2022) [24]. In Step 1, we set the intake success rate, which is the proportion of individuals with hypertension who are likely to incorporate flaxseed into their diet. In Step 2, we measure the reduction in SBP and DBP resulting from consuming flaxseed. In Step 3, we estimate the prevalence of hypertension in Canada and the reduction in this prevalence based on the effectiveness of flaxseed supplementation in reducing BP. Finally, in Step 4, we estimate the potential reduction in health care costs associated with the decrease in the prevalence of hypertension in Canada. Steps 2 to 4 are conducted using Monte Carlo simulation to account for uncertainty in the model parameters. All analyses were performed using R statistical software, version 4.3.2. A more detailed look at each step is presented below.

#### 2.1.1. Step 1: Intake Success Rate

In our first step, we set the dietary intake success rate. The dietary intake success rate represents the proportion of the population likely to adopt the flax-based regimen. While consumption and frequency data are the best sources to determine which success rates to use in our analysis, such data are not readily available, rendering these rates challenging to measure. In Canada, the only available data on flaxseed are the quantity produced, the quality of the grain, and the oil and protein content. In 2021, Canada produced 345,100 tonnes of flaxseed, down 40.3% from the levels in 2020, with lower oil and higher protein content than those found in the 2020 samples [25]. 

It is difficult, however, to distinguish between the quantity of dietary flaxseed produced and the quantity produced for other purposes, e.g., industrial purposes and animal feed. Furthermore, data regarding the human consumption of flaxseed in Canada are nonexistent. Therefore, we employ partial sensitivity analysis, selecting a range of intake success rates varying from pessimistic to optimistic, i.e., 5% (very pessimistic), 10% (pessimistic), 25% (optimistic), and 50% (very optimistic). By selecting multiple rates, we cover different scenarios of how the population alters its diet, given the newly available information. This is a common approach used in COI analyses [24,26,27,28].

#### 2.1.2. Step 2: Effect of Flaxseed on Blood Pressure

The second step of our analysis is to measure the effect of consuming flaxseed on SBP and DBP. Flaxseed contains several cardiovascular health-promoting bioactive compounds, including ALA omega-3 fatty acids, lignans, dietary fiber, protein, and potassium, which may improve circulation and reduce blood pressure [29]. These nutrients possess anti-inflammatory properties and have been demonstrated to reduce cholesterol levels, regulate blood sugar, decrease oxidative stress, and enhance nitric oxide production [12,13,14,15,16,17,18,21,22]. These effects contribute to blood vessel relaxation, which can aid in lowering blood pressure [17]. Ground flaxseed is typically preferred over whole flaxseed because grinding increases nutrient bioavailability [12,18]. The ground flaxseed can be added to various meals, including smoothies, oatmeal, yogurt, baked goods, salads, and soups. 

The hypotensive effects of flaxseed consumption have been thoroughly investigated through randomized control trials (RCTs) and meta-analyses, although the magnitude of the effect has been inconsistent [12,30,31,32,33,34,35,36]. Doses of 10 g to 100 g of flaxseed per day have been considered in RCTs, with daily doses of 30 g or more yielding a greater effect for lowering BP [12,16,18]. Most recently, Li et al. (2023) [12] performed a meta-analysis of thirty-three RCTs, with flax supplementation periods longer than two weeks. They found that flaxseed supplementation resulted in a weighted mean reduction of 3.19 (95% CI: 2.24, 4.15) mm Hg in SBP and 2.16 (95% CI: 1.94, 3.27) mm Hg in DBP. In our COI analysis, we assume that adopting the flax-based treatment will lead to a $U\left(2.24,4.15\right)$ mm Hg reduction in SBP and a $U\left(1.94,3.27\right)$ mm Hg reduction in DBP, where $U\left(\cdot \right)$ represents the uniform distribution. That is, to introduce uncertainty into the model, we randomly draw values from uniform distributions which cover the ranges defined by the 95% confidence intervals for these SBP and DBP measurements.

#### 2.1.3. Step 3: Hypertension Prevalence Reduction

We used data from the CHMS, Cycles 5 and 6, to estimate the potential reduction in hypertension. The CHMS collects health information on Canadians to develop an accurate picture of the physical well-being of the population. There are two components of the CHMS: the household questionnaire and the clinic questionnaire. The household component of the survey collects self-reported data on current and past health status, health-promoting behaviors, and chronic disease risk factors. In the clinical portion of the CHMS, physical measurements, including anthropometry, blood pressure, heart rate, physical fitness, and oral health, are recorded at a mobile examination center. Data from Cycles 5 and 6 of the CHMS were collected from January 2016 to December 2017 and from January 2018 to December 2019, respectively. The weights used in the analysis are those provided for the linked file. The study population of interest is individuals 18 years or older who participated in the CHMS clinical component.

To estimate the prevalence of hypertension in Canada, we first computed the weighted averages of SBP and DBP for individuals in the study population. Next, we defined hypertension as anyone with an SBP over 140 mm Hg, a DBP over 90 mm Hg, and/or self-reported use of medications for hypertension. We then generated the variable Hypertension, which took a value of 1, if a respondent met the definition for hypertension, and zero otherwise. That is, for individual i in the study population S:

(1) $$Hypertension_{i}=\left\{\begin{array}{ll} 1 & \mathrm{if}\;SBP_{i}\geq 140\;\mathrm{mm}\;\mathrm{hg},\;DBP_{i}\geq 90\;\mathrm{mm}\;\mathrm{Hg},\\ 0 & \mathrm{and}/\mathrm{or}\;\mathrm{takes}\;\mathrm{medication}\;\mathrm{for}\;\mathrm{hypertension}\\ 0 & \mathrm{otherwise} \end{array}\right.$$

The crude prevalence of hypertension was then calculated as the weighted average of the hypertension variable:

$$\mathrm{Prevalence}=\frac{\sum_{i=1}^{n_{S}}Hypertension_{i}\times w_{i}}{\sum_{i=1}^{n_{S}}w_{i}},$$

where $n_{S}$ is the sample size and $w_{i}$ is the survey weight for individual i. To estimate the reduction in the prevalence of hypertension, we take a random subsample of hypertensive individuals, based on the intake success rate. These are the theoretical individuals who adopt the flax-based treatment. We then subtract the expected effect of the flax-based treatment from the SBP and DBP for each respondent in the subsample. If the individual’s SBP and DBP fall below the hypertension thresholds, the individual is no longer considered hypertensive in our model. 

A challenge we encountered in our data was that some individuals already taking medication for hypertension exhibited SBP and DBP values below the hypertension thresholds. Therefore, we adjusted these values by removing the expected effect of a standard dose of hypertensive medications before our analysis. According to estimates from Law et al. (2003) [37], a standard dose of hypertensive medication decreases SBP by 9.1 mm Hg (95% CI: 8.8, 9.3) and DBP by 5.5 mm Hg (95% CI: 5.4, 5.7). For individuals currently on anti-hypertensive medications, we added random draws from $U\left(8.8,9.3\right)$ and U(5.4,5.7) to their SBP and DBP, respectively.

#### 2.1.4. Step 4: Potential Health Care Cost-Savings

To calculate the potential cost-savings from the flax-based treatment, we first estimate the per capita health care costs related to hypertension in Canada. For this calculation, we used results from Weaver et al. (2015) [11], who used administrative data from the province of Alberta to estimate the health care costs attributed to hypertension in Canada in 2020. Their country-wide estimates are based on the assumption that overall Canadian health care costs are 17% lower than those in Alberta. In our analysis, we assume that health care costs are 4.27% greater in Alberta compared to those for all of Canada, based on the latest data from the Canadian Institute of Health Information [38]. Table 1 displays the estimated per capita health care costs associated with hypertension in Canada in 2020, presented in 2020 Canadian dollars (CAD) for each age group. Unfortunately, the costs are not broken down by gender. Therefore, we used the same costs for men and women in the same age group.

Given the intake success rate, the effect of consuming flaxseed on blood pressure, the SBP and DBP measurements in the CHMS, and the estimated per capita health care costs, we estimated the reduction in health care costs from a decrease in the prevalence of hypertension in 2020. In our analysis, we assume that the prevalence of hypertension remains stable between 2019 and 2020.

### 2.2. Simulations

#### 2.2.1. Notation

Let H denote the subset of individuals who meet the definition for hypertension. Each i∈H represents $w_{i}$ individuals in the population, where $w_{i}$ is the survey weight for individual i. We begin our analysis by expanding the dataset by repeating $w_{i}-1$ observations for each i∈H. Let $H^{\prime }$ denote the expanded sample. In total, we have $n_{H^{\prime }}=\sum_{i=1}^{n_{H}}w_{i}$ observations, where $n_{H}$ is the number of observations in H. The total cost attributable to hypertension can be calculated as:

$$\mathrm{Total}\;\mathrm{Cos}t=\sum_{i=1}^{n_{H^{\prime }}}C_{i},$$

where $C_{i}$ is the cost attributable to hypertension for individual $i\in H^{\prime }$ and is based on the values in Table 1. $H^{\prime }$ can be divided into two groups, $H^{\prime }=W+M$, where W is the subset of individuals who do not take medication for hypertension, and M is the subset of individuals who take medication for hypertension.

#### 2.2.2. Sensitivity Analysis

To incorporate uncertainty into the cost of illness (COI) analysis, we employed a combination of partial sensitivity analysis and Monte Carlo simulations. Partial sensitivity analysis involves repeating the analysis, while varying a single model parameter. In our study, we applied this method to vary the intake success rate at 5%, 10%, 25%, and 50%. Monte Carlo simulation is a robust analytic tool that simultaneously varies multiple model parameters by drawing values from specified distributions. This approach allows for a more comprehensive assessment of the model’s sensitivity to parameter changes and enhances the reliability of our findings. For Steps 2–4 of our COI, we employ a Monte Carlo simulation, with each iteration comprising the following steps:

1. For each j∈M, we adjust $SBP_{j}$ and $DBP_{j}$ to remove the effects of a standard dose of medications for hypertension. Let $SBP_{j}^{Adj}$ and $DBP_{j}^{Adj}$ denote the adjusted SBP and adjusted DBP, respectively, for individual j∈M. Then:
  $$SBP_{j}^{Adj}=SBP_{j}+B_{j},B_{j}∼U\left(8.8,9.3\right)$$
  and
  $$DBP_{j}^{Adj}={DBP}_{j}+D_{j},\;{\;D}_{j}∼U\left(5.4,5.7\right),\;$$
  where $U\left(\cdot \right)$ denotes a random draw from the uniform distribution. For individual $i\in H^{\prime }$ and i∉M, we set ${SBP}_{i}^{Adj}={SBP}_{i}$ and ${DBP}_{i}^{Adj}={DBP}_{i}$.
2. Let K denote the subset individuals with ${SBP}^{Adj}\geq 140\;\mathrm{mm}\;\mathrm{Hg}$ and/or $DBP^{Adj}\geq 90\;\mathrm{mm}\;\mathrm{Hg}$. From K, we take a random sample of size $r\times n_{K}$, where r is the intake success rate and $n_{K}$ is the number of individuals in K. These are the individuals with hypertension who theoretically take the flax-based treatment.
3. Let F denote the $r\times n_{K}$ sample in 2. For each l∈F, we estimate the effectiveness of the flax-based treatment. To estimate the impact of the flax-based treatment on SBP, we subtract a random variable $U_{l}^{S}$ from ${SBP}_{l}^{Adj}$ for each l∈F, where $U_{l}^{S}$ is a random draw from $U\left(2.24,4.15\right)$. To estimate the impact of the flax-based treatment on DBP, we subtract a random variable $U_{l}^{D}$ from the ${DBP}_{l}^{Adj}$ for each l∈F, where $U_{l}^{D}$ is a random draw from $U\left(1.94,3.27\right)$. Let ${SBP}_{l}^{Flax}$ and ${DBP}_{l}^{Flax}$ denote the new SBP and DBP, respectively, after the flax-based treatment for individual l. Then:
  $${SBP}_{l}^{Flax}={SBP}_{l}^{Adj}-U_{l}^{S}$$
  and
  $${DBP}_{l}^{Flax}={DBP}_{l}^{Adj}-U_{l}^{D}.$$
4. We generate a new hypertension variable, which takes a value of 1 if the individual has an SBP ≥ 140 mm Hg and/or a DBP ≥ to 90 mm Hg. We call this variable ${Hypertension}^{New}$. For individual l∈F:
  $${Hypertension}_{l}^{New}=\left\{\begin{array}{ll} 1 & \mathrm{if}\;{SBP}_{l}^{Flax}\geq 140\;\mathrm{mm}\;\mathrm{Hg}\;\mathrm{and}/\mathrm{or}\;{DBP}_{l}^{Flax}\geq 90\;\mathrm{mm}\;\mathrm{Hg}\\ 0 & \mathrm{otherwise}. \end{array}\right.$$
  For individual i∈H, but i∉F, we set ${Hypertension}_{i}^{New}={Hypertension}_{i}.$
5. We calculate the reduced total health care costs associated with hypertension as
  $$\mathrm{Adjusted}\;\mathrm{Cos}\mathrm{ts}=\sum_{i=1}^{n_{H^{\prime }}}{Hypertension}_{i}^{new}\times C_{i}.$$
6. We estimate the potential health care cost-savings as:
  Health Care Savings=(Total Costs)−(Adjusted Costs).

Finally, we run 1000 iterations for each intake success rate and present the mean results.

## 3. Results

### 3.1. Prevalence of Hypertension

**Figure 3:**
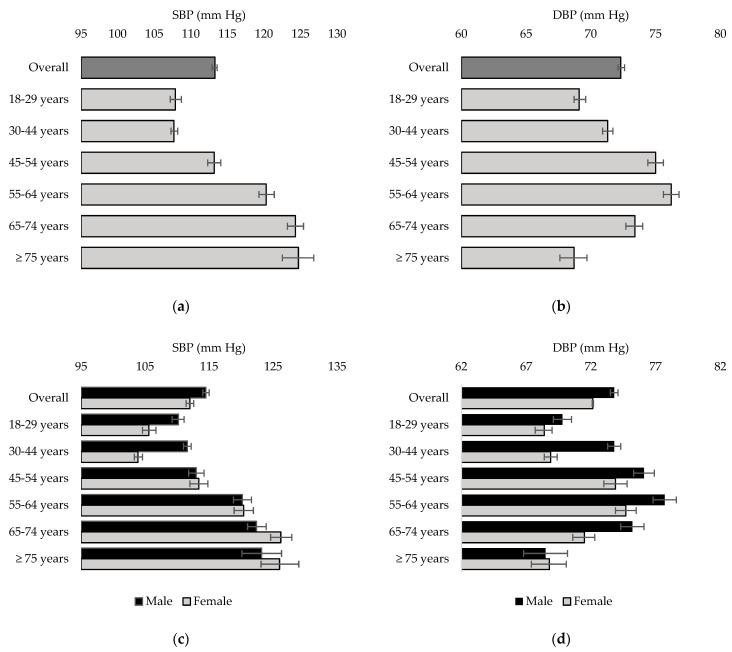
Average SBP and DBP, by sex and age group, for the household population aged 18 years and older (error bars represent the 95% confidence interval). (**a**) Average SBP for both sexes; (**b**) average DBP for both sexes; (**c**) average SBP, male vs. female; (**d**) average DBP, male vs. female.

Figure 3 displays the results for the average SBP and average DBP for Canadians 18 years or older, respectively. Overall, the average SBP and DBP were 113.3 mm Hg (95% CI: 112.9, 113.6) and 72.3 mm Hg (95% CI: 72.1, 72.6) for both sexes. While the average SBP increased for older age groups, the average DBP was the highest for the 55 to 64 years age group (76.2; 95% CI: 75.6,76.8). Overall, males had a higher SBP and DBP compared to females, with males exhibiting a higher DBP for ages 18 to 74. 

**Figure 4:**
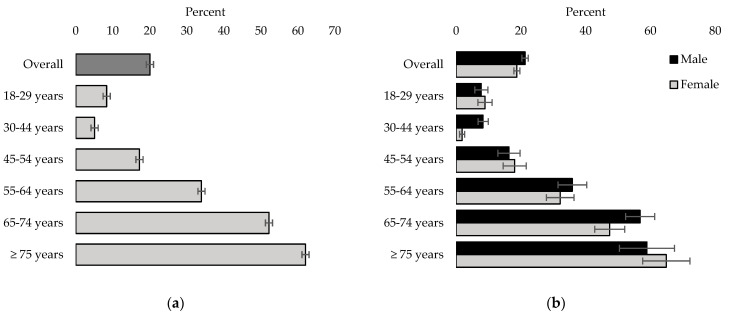
Estimated prevalence of hypertension in Canada, 2016–2019, by sex and by age group, household population 18 years and older (error bars represent the 95% confidence interval). (**a**) Both sexes; (**b**) male vs. female.

Figure 4 displays the estimated prevalence rates of hypertension in Canada by age group. The estimated prevalence of hypertension in Canadians 18 years or older is 20.02% (95% CI: 19.06%, 20.99%). The prevalence is highest for individuals 55 years and older, with 62% (95% CI: 61.09, 63.01) of individuals 75 years or older meeting the criteria for hypertension (Figure 4a). Males (21.31%, 95% CI: 20.33, 22.29) exhibit a higher prevalence overall compared to that of females (18.75%, 95% CI:17.81, 19.68).

### 3.2. COI Analysis

The potential health care cost-savings associated with using flaxseed to decrease the prevalence of hypertension are summarized in Table 2. These are the Monte Carlo estimates for each intake success rate. Under the very optimistic scenario, assuming a 50% success rate, our analysis predicted annual health care cost-savings equal to CAD 985.2 M (95% CI: 970.3 M, 1 B) in 2020. Assuming a 25% success rate, the optimistic scenario predicted savings of CAD 490.4 M (95% CI: 474.8 M, 506 M) for individuals 18 years and over. With a 10% success rate, the pessimistic scenario showed a cost-savings of CAD 195.1 M (95% CI: 178.2 M, 212.1 M). Finally, the very pessimistic scenario of a 5% success rate suggested a total savings of CAD 96.3 M (95% CI: 79.4 M, 113.1 M).

## 4. Discussion

**Figure 5:**
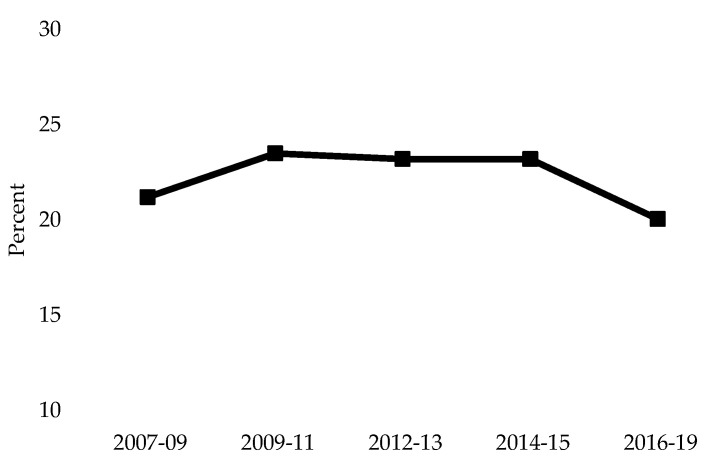
Estimated crude prevalence of hypertension in Canada from Cycles 1–6 of the Canadian Health Measures Survey.

**Figure 6:**
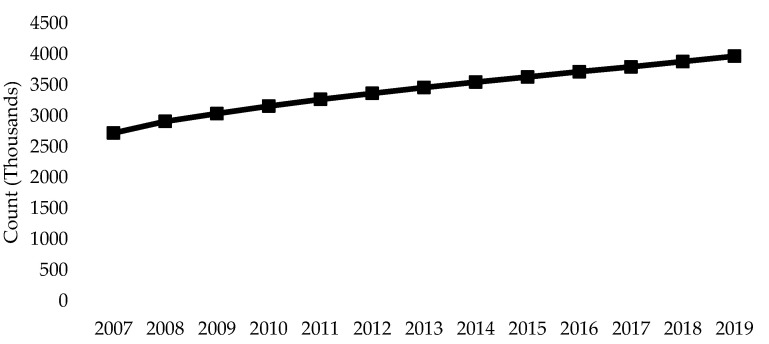
Population count of hypertension in Canada, both sexes, 20 years and older (source: Canadian Chronic Disease Monitoring System (2021) [39]).

Our results indicate that approximately one in five Canadians aged 18 years or older have hypertension. These findings align with previous estimates of hypertension prevalence in Canada and even suggest a slight decrease compared to earlier cycles of the CHMS (Figure 5). While this is encouraging, the overall population has been increasing, with the population of Canada surpassing 40 million people on June 16, 2023 [2]. Consequently, although the prevalence of hypertension has remained stable over recent years, the absolute number of individuals affected by this condition is rising (Figure 6) [39]. This trend further exacerbates the burden of hypertension on the Canadian health care system.

Our four-step COI analysis yielded promising estimates for the potential cost-savings from a reduced prevalence of hypertension due to flaxseed consumption. Even under the most pessimistic scenario, we estimated a reduction in health care costs of approximately CAD 96 million in 2020. With the implementation of appropriate policies, adjustments to the Canadian Food Guide, and education of health care professionals, potential annual savings could reach up to CAD 986 million, if 50% of the targeted population adopts this dietary regimen. These figures do not account for the additional health benefits and cost-savings associated with dietary flaxseed’s capacity to reduce plasma cholesterol and LDL by 10–15% [21]. Decreased cholesterol levels would likely result in fewer significant cardiac events and strokes [21], further reducing health care costs.

These estimates provide a strong argument for legislators to implement policies promoting flaxseed as a treatment for hypertension and cholesterol reduction. Such policies will likely encounter minimal resistance, as they redirect government expenditure towards diseases lacking alternative therapies or other social programs. Moreover, the successful implementation of flaxseed-related policies could spur further research into the use of functional foods as alternative therapies for various diseases, amplifying the benefits of this research. 

Despite the evidence suggesting significant health care cost-savings from flaxseed treatment, the potential unintended consequences must be considered. Given that hypertension is the leading global risk factor for death and frequently accompanies major causes of hospitalization and mortality in Canada, it is reasonable to expect that a substantial proportion of individuals will experience increased life expectancy [3,40]. This extended lifespan could lead to higher lifetime health care expenditures, which might offset the cost-savings from flaxseed treatment. However, as noted by Abdullah et al. (2017) [26], the potential increase in quality-adjusted life years provides a compelling reason to pursue strategies to reduce chronic disease.

Public perception of alternative medications, particularly dietary interventions, often focuses on potential negative aspects. While flaxseed might be associated with minor issues, like increased bowel movements or temporary digestive discomfort, it generally lacks significant negative public opinion. Concerns about phytoestrogens, cadmium, or protease inhibitors in flaxseed are either unsubstantiated [19] or not widely recognized [41]. With adequate education for health care professionals and the implementation of appropriate policies, professionals can effectively promote flaxseed use and provide patients with accurate information about potential complications.

There are, of course, other nutritional interventions that have been shown to reduce hypertension. The reduction in salt intake identified in the low sodium DASH Trial [42] showed that this reduction can substantially reduce BP. A diet that includes plenty of vegetables, fruits, and low-fat dairy foods and includes a lower total and saturated fat content can significantly reduce BP in hypertensive individuals [43]. Diets rich in potassium, polyunsaturated fatty acids, and protein may also reduce BP [44]. Obesity also contributes to an elevation in BP, and weight reduction effectively lowers BP [44]. Increasing the frequency of exercise may also be an effective behavioral change which can lower BP [44]. Additionally, reducing alcohol consumption can have a significant effect on BP [43]. Two recent studies have also shown that team-based care for hypertension may be more successful and cost-effective for reducing BP [45,46]. Chay et al. (2024) [45] estimated the cost per unit reduction in disability-adjusted life years (DALY) for a multi-component primary care approach to treating hypertension, which included clinical training in hypertension management, subsidized medications, nurse consultations, and telephone follow-ups. This approach successfully reduced BP and was deemed cost-effective, based on a threshold of CAD 55,500 per DALY. Bryant et al. (2023) [46] found that using team-based care (≥ 2 team members) to reduce BP yielded significant reductions in BP compared to those obtained from usual care, and this method is cost-effective at CAD 4400 per quality-adjusted life year.

This study was the first to assess the potential health care cost-savings from consuming flaxseed to reduce BP. We used the latest cycles of the CHMS to estimate the prevalence of hypertension in Canada, and we used the micro-data measures of SBP and DBP to estimate the individual effect of consuming flaxseed on BP. Despite the strengths of this study, the following limitations must be acknowledged. First, we simplified the analysis by removing the effect of hypertension medications on BP. We used an “overall” effect and did not consider the effect of individual medication types (e.g., diuretics, ACE inhibitors, calcium channel blockers, beta-blockers, or their combinations). We used a meta-analysis by Law et al. (2003) [37] to measure the overall effect of hypertension medications on BP, as pooling results from multiple RCTs lowers the probability that the estimate will be negatively affected by the limitations of one study. Second, there are limitations in what we understand about the benefits of a flax-enriched diet for health in general and for cardiovascular health specifically. This limitation has been identified in detail elsewhere [19]. For example, the lowest effective dose of flaxseed to lower BP has not yet been determined in a controlled dose-dependent investigation. Therefore, this dosage needs to be studied. With regard to mechanism, it remains unclear whether flaxseed can inhibit the metabolism of anti-hypertensive drugs and thereby achieve an additional hypotensive effect. In addition, although supplementation of the diet with flaxseed lowered BP in patients with peripheral arterial disease, it provided no improvement regarding the capacity of the patients to exercise [47]. Much remains to be studied about the health benefits of dietary flaxseed.

## 5. Conclusions

Our research suggests that economic or financial modeling can be useful in providing incentives and evidence for the adoption of alternative forms of medication, specifically the use of dietary flaxseed to treat hypertension. The resulting estimates from our study should garner attention from both provincial and federal government levels. This attention could drive the implementation of educational policies to inform healthcare professionals and further promote the benefits of flaxseed to the public. 

## Figures and Tables

**Table 1:** Annual health care costs attributable to hypertension in Canada in 2020.

Age Group	Per Capita Costs ^1^ (CAD)
18–44 years	2095.73
45–54 years	2305.30
55–64 years	2514.87
65–74 years	2934.02

^1^ Estimates based on 2020 per capita public health expenditures being 4.27% greater in Alberta than in Canada as a whole (CAD 7014.33 vs. CAD 6727.38, excluding expenditures for public health initiatives and COVID-19 measures).

**Table 2:** Potential annual health care cost-savings from flax-based treatment for hypertension under varying intake success rates, CAD millions, 2020 Canadian dollars (95% confidence interval).

Intake Success Rate (%)	Estimated Cost-Savings (CAD)
5	96.28 (79.44, 113.12)
10	195.12 (178.17, 212.07)
25	490.39 (474.78,506)
50	985.15 (970.31, 999.98)

## Data Availability

Data for this project were accessed through the Canadian Research Data Center Network. The R code is available upon request.
